# Proportion of contextual effects in the treatment of fibromyalgia—a meta-analysis of randomised controlled trials

**DOI:** 10.1007/s10067-017-3948-3

**Published:** 2017-12-20

**Authors:** Nicola Whiteside, Aliya Sarmanova, Xi Chen, Kun Zou, Natasya Abdullah, Michael Doherty, Weiya Zhang

**Affiliations:** 1 Academic Rheumatology, School of Medicine, University of Nottingham, Clinical Sciences Building, Nottingham City Hospital, Nottingham, NG5 1PB UK; 2 Haihe Pharmaceutical Co., Ltd., Shanghai, China; 3 Department of Medical Record and Statistics, Sichuan Academy of Medical Sciences and Sichuan Provincial People’s Hospital, Affiliated Hospital of The University of Electronic Science and Technology, Chengdu, China

**Keywords:** Contextual effect, Fibromyalgia, Meta-analysis, Systematic review

## Abstract

The objective of this study is to examine the proportion of the total treatment effect that is attributable to contextual effects in randomised controlled trials (RCTs) of treatments for fibromyalgia. A systematic literature search was undertaken in Medline, Web of Science, EMBASE, Cumulative Index to Nursing and Allied Health Literature (CINAHL), and Allied and Complementary Medicine in September 2015. The proportion of contextual effect (PCE) was calculated by dividing the improvement in the placebo arm by the improvement in the treatment arm. The measure was log-transformed for each trial and the random effects model was used to pool data. The primary outcome was pain. Secondary outcomes were fibromyalgia impact questionnaire (FIQ) total and fatigue. Heterogeneity was quantified using *I* ^2^. Publication bias was assessed using a funnel plot and Egger’s test. Subgroup analysis was undertaken to explore heterogeneity and potential determinants of the PCE. Fifty-one eligible trials (9599 participants) were identified. The PCE was 0.60 (95% CI 0·56 to 0·64) for pain, 0·57 (95% CI 0·53 to 0·61) for FIQ total, and 0·63 (95% CI 0·59 to 0·68) for fatigue. The *I* ^2^ was 99.4% for pain, 99.2% for FIQ total, and 97.6% for fatigue. More than half of the treatment effect in fibromyalgia RCTs results from non-specific contextual factors. This suggests that optimising contextual care may enhance treatment effects and improve outcomes. Reporting the total treatment effect and the proportion of contextual effect in trials may help to better translate research evidence into practice.

**Electronic supplementary material:**

The online version of this article (10.1007/s10067-017-3948-3) contains supplementary material, which is available to authorized users.

## Introduction

Fibromyalgia is a chronic painful and distressing condition with an estimated worldwide prevalence of 2.7% [1]. Recognised risk factors include female sex, increasing age, poor socioeconomic status, low education, and residence in rural areas [1]. The main symptoms are multiple regional musculoskeletal pain, stiffness, non-restorative sleep, fatigue, distress, and cognitive difficulties. Demonstrable abnormalities include widespread hyperalgesia (reduced pain threshold), allodynia, and reduced delta sleep [2]. Currently, there is no cure and the aim of treatment is to relieve symptoms and improve quality of life. An individualised multidisciplinary approach combining patient education, non-pharmacological and pharmacological treatments is recommended [3, 4].

Fibromyalgia treatments have been extensively evaluated in randomised controlled trials (RCTs). A meta-analysis of eight interventions (including tricyclic antidepressants, selective serotonin reuptake inhibitors, serotonin noradrenaline reuptake inhibitors (SNRIs), the gamma-amino butyric acid analogue pregabalin, aerobic exercise, balneotherapy, cognitive behavioural therapy (CBT), and multicomponent therapy) found that when lower quality studies are excluded the effect for pharmacological treatment becomes small and there is little evidence for effect from non-pharmacological treatments [5]. When studies with less than 100 participants in each arm were excluded the difference between treatments and placebo was judged to be too small to be clinically relevant.

The main focus in current reporting of RCTs is on the separation of the treatment group from the placebo and a treatment is deemed effective only if it is significantly better than placebo. That is, the judgement for treatment efficacy is based solely on the specific treatment effect. However, the benefit that a patient experiences derives not only from the specific effects of the treatment but also from the non-specific effects of the context in which the treatment is delivered [6]. This creates an ‘efficacy paradox’ when a patient in a clinical setting experiences significant benefit from a treatment that has been deemed ineffective in an RCT because it did not separate sufficiently from placebo [6]. This paradox is common with fibromyalgia, a condition that associates with a significant placebo effect in RCTs [7]. Therefore, to better reflect patient-centred experience, instead of evaluating a treatment solely on the difference between treatment and placebo (specific effect), we propose to additionally present the total treatment effect and the proportion attributed to contextual effects (PCE). The concept of PCE has been previously evaluated for the treatment of osteoarthritis and depression [6, 8]. Both studies revealed that the majority of the total treatment effects were contextual (exhibited in the placebo arm). The aim of this study was to examine the PCE in the context of the total treatment effect using the RCT data in fibromyalgia.

## Methods

A systematic review and meta-analysis was carried out according to the Preferred Reporting Items for Systematic Reviews and Meta-Analyses (PRISMA) guidelines [9].

### Data sources and searches

Electronic databases were used for the literature search, specifically Medline (1950–), Web of Science (1960–), EMBASE (1980–), Cumulative Index to Nursing and Allied Health Literature (CINAHL) (1982–), and Allied and Complementary Medicine (1985–). For Medline, both PubMed and OVID databases were searched. The search was undertaken initially in December 2014 and updated in September 2015 to identify new studies. There were no language restrictions. All results from the database search were exported into EndNote and duplicates were removed. Initially, those with clearly irrelevant titles or abstracts were excluded. If papers could not be excluded via their abstract, full-text copies were obtained and evaluated.

### Study selection

**Fig. 1:**
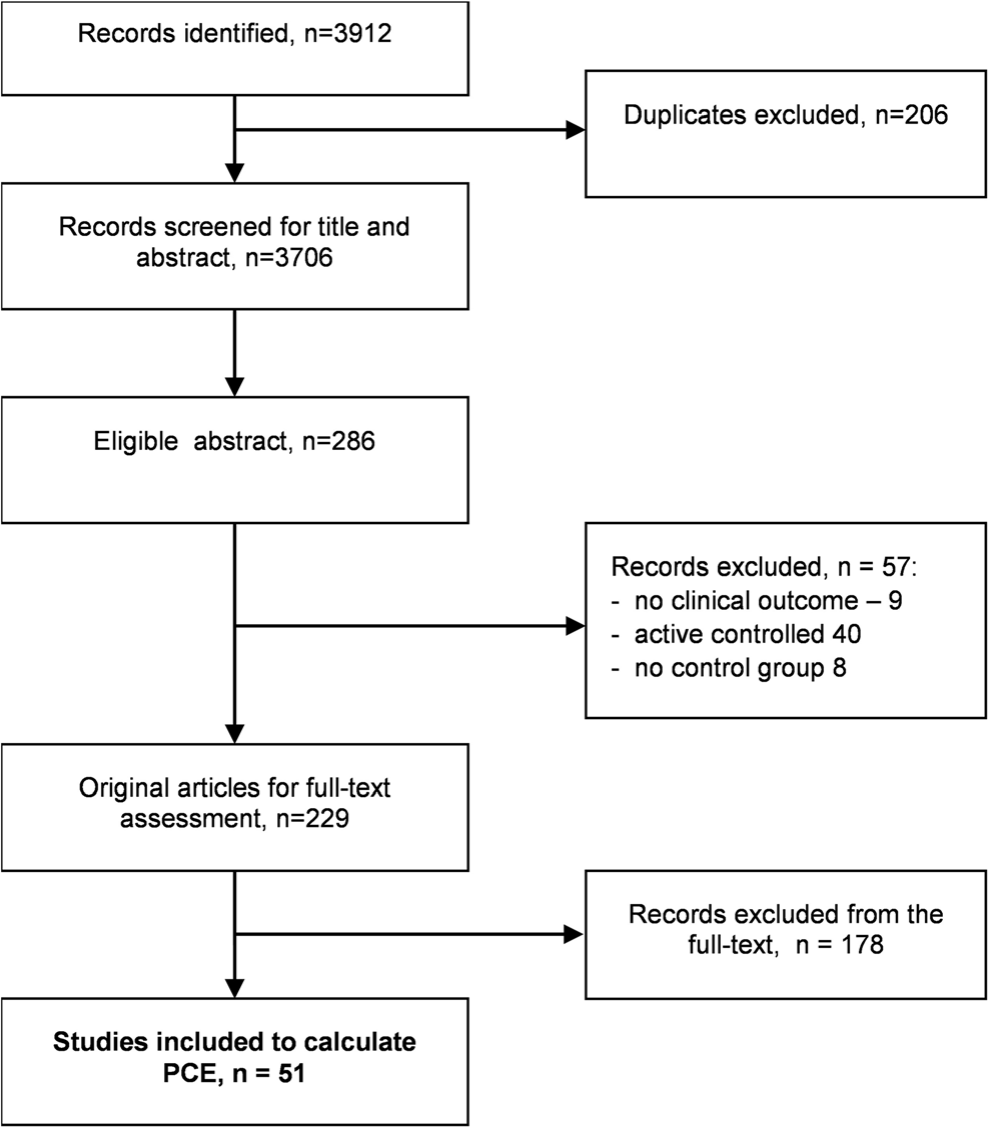
Study selection

There were no age, gender, or ethnicity restrictions. There were also no restrictions on dosage, frequency of delivery, duration of delivery, mode of delivery, or timing of delivery. Studies with relevant clinical outcomes were included, specifically those reporting the Beck Depression Inventory, fatigue score, Fibromyalgia Impact Questionnaire (FIQ) total, visual analogue scale (VAS) pain scale, number of tender points, physical function, and sleep quality. Studies with non-clinical outcomes were not included (Fig. 1).

Only placebo-controlled trials were included to calculate PCE in our main analysis. Any trial in which the treatment or placebo group outcomes worsened or did not change were discarded when calculating the PCE as the negative or zero PCE would not permit log transformation for the meta-analysis [10].

#### Treatment categories

All medications were categorised according to the British National Formulary (https://www.bnf.org/). These included antidepressants (e.g. duloxetine, amitriptyline, fluoxetine, esreboxetine, citalopram), fibromyalgia agents (e.g. milnacipran), anticonvulsants (e.g. gabapentin and pregabalin) and analgesics (e.g. tramadol, acetaminophen/paracetamol, carisoprodol, and caffeine). All remaining medications were merged into one category as ‘others’ (dolasetron, tropisetron, Nutropin, dehydroepiandrosterone, terguride, pyridostigmine, cyclobenzaprine, interferon alpha). Non-pharmacological treatments included acupuncture, electromagnetic therapy, and other treatment modalities.

### Data extraction

Data were extracted by the first reviewer (NW) and validated independently by a second reviewer (AS). Discrepancies were discussed between the two reviewers and any disagreement was discussed with a third reviewer (WZ). The following data were extracted: country in which trial was performed, setting (e.g. community or hospital), number of arms, trial design, presence of industry funding, treatment, type of blinding, presence of allocation concealment, use of intention to treat analysis, duration of treatment, frequency/dose of treatment, diagnosis method, continuation of existing therapy, age, proportion of female participants, duration of fibromyalgia, number of participants, dropout rate, outcome measure description, and results with their standard deviations.

### Quality assessment

The RCTs were assessed using the adapted Jadad scale [11]. Allocation concealment (yes/no/unknown) and blinding to patient, physician, and assessor (yes/no) were added in the assessment form to counter the caveats of the Jadad scale.

### Data synthesis and analysis

Baseline score, post-intervention score, and change from baseline were entered into the database with their standard deviations. Calculations were undertaken if needed to obtain all these values based on the information provided.

PCE was calculated based on the following formula:

$$ \mathrm{PCE}=\frac{\mathrm{Improvement}\  \mathrm{in}\  \mathrm{placebo}\  \mathrm{group}}{\mathrm{Improvement}\  \mathrm{in}\  \mathrm{treatment}\  \mathrm{group}} $$

where the numerator presents the contextual effect and the denominator presents the contextual plus specific effects, i.e. total treatment effect. PCE was log-transformed to normalise the distribution. Standard error (SE) of the log(PCE) was calculated using the Hedges method [12]. The standardised mean change from baseline for the treatment group was calculated to present the total treatment effect. In order to calculate the PCE, only improvement of the outcome (e.g. pain relief) was considered. No improvement from baseline (change score = 0) or worsening (negative score) from baseline will be excluded from this calculation. This is because the zero change score or negative score does not permit log transformation of the PCE and it is not a beneficial outcome relevant to the aims of calculating the PCE. It also cannot explain why a treatment worsens the targeted beneficial outcomes unless it is by chance or an error. Therefore, we excluded them from the PCE calculation. A random effects model was used to pool the results with STATA, and the outcomes were stratified according to treatment and other subgroup indicators. I^2^ index was used to assess heterogeneity [13]. Publication bias was examined using the funnel plot and Egger’s test [14]. Meta-regression analysis (random effects regression) was undertaken if any subgroups showed substantial heterogeneity. The PCE was presented together with the total treatment effect in proportions to demonstrate both contributions from the specific treatment and non-specific contextual effects.

#### Data availability statement

The dataset(s) supporting the conclusions of this article are available on request from the corresponding author.

## Results

### Selection of studies

In total, 3912 citations were retrieved from the systematic literature search. Two hundred six duplicates were removed. Two hundred eighty-six potentially eligible studies were identified from reading the abstracts. Full texts were obtained for all 286 papers and 51 gave data for the PCE. A full list of the included studies can be found in Appendix 1.

### Characteristics of included studies

Of the 51 studies included in the primary analysis, 46 investigated pain, 30 investigated FIQ total, and 27 investigated fatigue (Table 1).

**Table 1:** Summary of characteristics of included studies

	Treatment	Placebo
No. of participants	4795	4804
Mean age (range), year	48.8 (39.4–59.2)	48.58 (35.3–58.7)
Mean percentage of women (range), %	91.7 (39.2–100)	91.7 (61.8–100)
Median years of symptom (range)	7.8 (2.9–16.1)	7.9 (3.3–14.4)
Mean duration of treatment (weeks)	12.2	
Jadad score, mean	4.47	
Industry funding, *n* (%)	26 (51.0)	
Dropout rate, *n* (%)	37 (72.5)	
Random sequence, *n* (%)	32 (62.7)	
Allocation concealment, *n* (%)	31 (61.0)	
Blinding participants, *n* (%)	47 (92.2)	
Blinding care provider, *n* (%)	34 (66.7)	
Blinding assessors, *n* (%)	38 (74.5)	
Intention to treat analysis, *n* (%)	26 (51.0)	

### Proportion of contextual effect

**Fig. 2:**
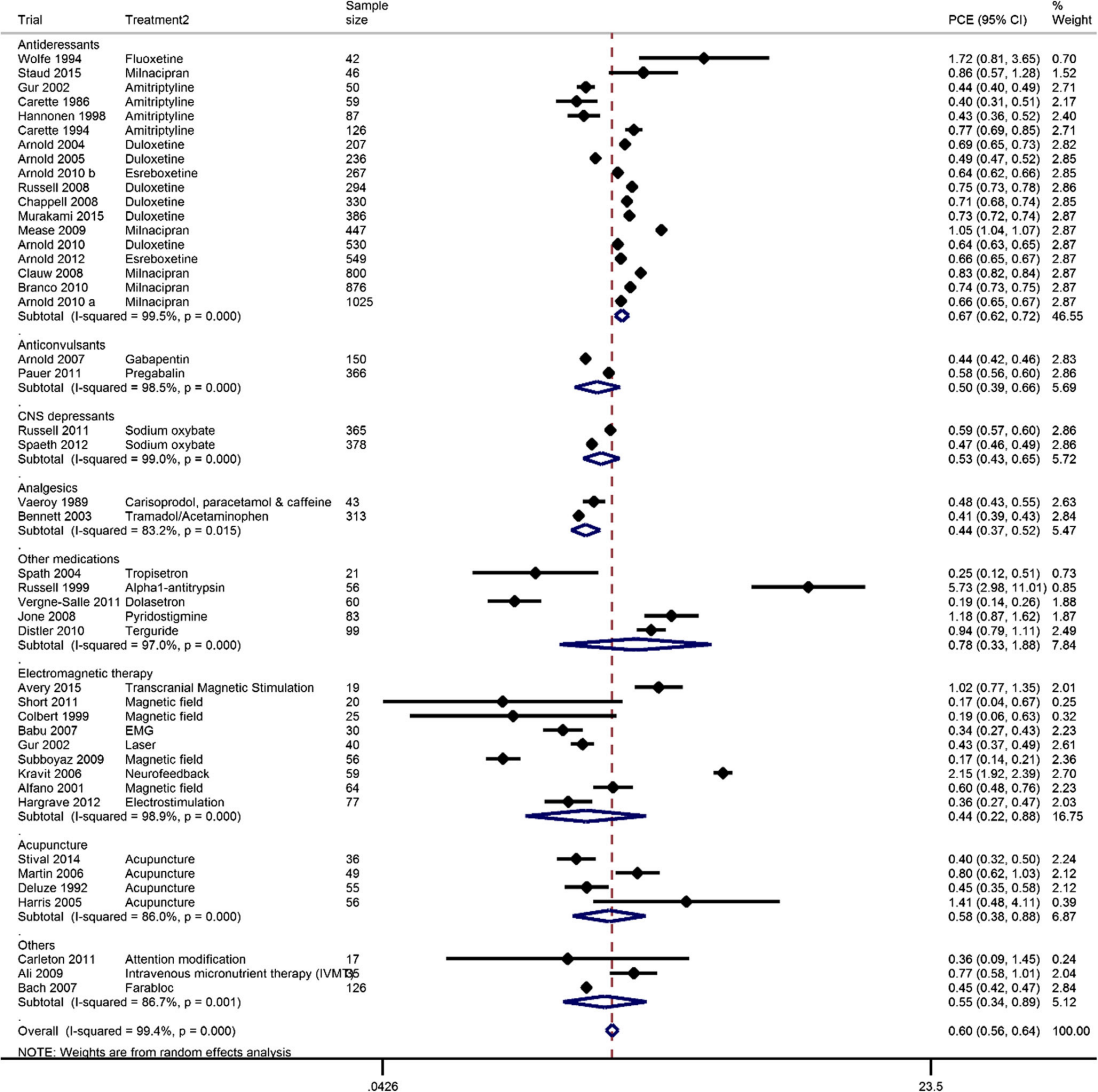
Forest plot of the proportion of contextual effect (PCE) for pain in fibromyalgia. PCE proportion of contextual effect, CI confidence interval, *I* ^2^ the variation in PCE attributable to heterogeneity, CNS central nervous system

The PCE for all treatments of fibromyalgia was 0.60 (95% CI 0·56 to 0·64) for pain, 0·57 (95% CI 0·53 to 0·61) for FIQ total, and 0·63 (95% CI 0·59 to 0·68) for fatigue score (Table 2). The heterogeneity between treatments varied according to the outcome measured: I^2^=99.4% for pain, I^2^=99.2% for FIQ total and I^2^=97.6% for fatigue. Publication bias was detected in studies for pain and FIQ total (*p* = 0.014 and *p* = 0.017, respectively). Figure 2 shows a forest plot for pain outcome.

**Table 2:** Summary of findings

	Number of studies included	Number of participants	PCE	95%CI	*I* ^2^ **,** %	*P* (Egger test)
Pain						
Antidepressants	19	6399	0.668	0.616 to 0.725	99.5	
CNS depressants	2	743	0.527	0.427 to 0.650	99.0	
Anticonvulsants	2	516	0.504	0.387 to 0.655	98.5	
Analgesics	2	356	0.440	0.371 to 0.520	83.2	
Other medications	5	319	0.782	0.326 to 1.880	97.0	
Electromagnetic therapy	9	390	0.442	0.221 to 0.885	98.9	
Acupuncture	4	196	0.577	0.379 to 0.879	86.0	
Others	3	178	0.548	0.339 to 0.888	86.7	
Overall	46	9097	0.599	0.557 to 0.643	99.4	*0.014*
FIQ total						
Antidepressants	11	4993	0.623	0.565 to 0.686	99.5	
Anticonvulsants	3	890	0.527	0.456 to 0.608	97.8	
CNS depressants	2	743	0.577	0.395 to 0.843	99.8	
Analgesics	1	313	0.555	0.533 to 0.578	–	
Other medications	4	292	0.687	0.502 to 0.940	90.5	
Electromagnetic therapy	7	265	0.379	0.239 to 0.599	94.7	
Acupuncture	1	49	0.634	0.482 to 0.835	–	
Others	1	35	0.537	0.384 to 0.749	–	
Overall	30	7580	0.569	0.530 to 0.612	99.2	*0.017*
Fatigue						
Antidepressants	12	4733	0.625	0.578 to 0.674	97.5	
Anticonvulsants	1	374	0.702	0.675 to 0.730		
CNS depressants	2	743	0.547	0.473 to 0.633	98.3	
Analgesics	1	313	0.808	0.773 to 0.845		
Other medications	2	135	1.272	0.449 to 3.601	59.7	
Electromagnetic therapy	6	263	0.396	0.221 to 0.708	90.3	
Acupuncture	2	105	1.075	0.230 to 5.028	97.4	
Others	1	62	0.330	0.165 to 0.661		
Overall	27	6728	0.632	0.588 to 0.680	97.6	0.820

*PCE* proportion of contextual effect, *CI* confidence interval, *I* ^2^ the variation in PCE attributable to heterogeneity, *CNS* central nervous system, *FIQ* Fibromyalgia Impact Questionnaire

**Fig. 3:**
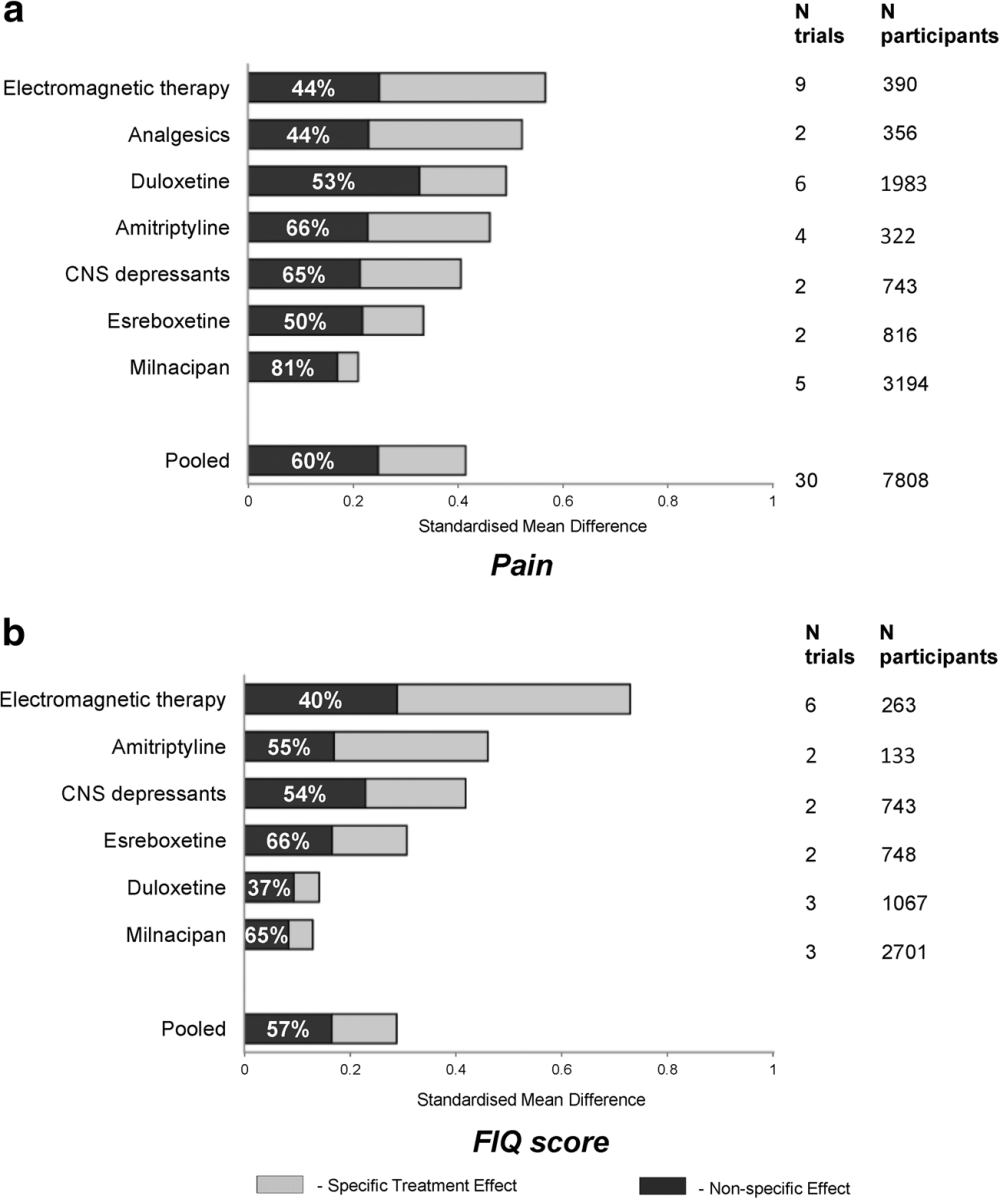
Total treatment effect and proportion of contextual effect in fibromyalgia trials

Figure 3 presents the total treatment effect for pain outcome and the proportion that is attributable to the contextual effect for all treatment modalities. The length of the bar represents the standardised total treatment effect for each treatment category. The percentage is the proportion of contextual effect (blue). Therefore, the remaining part of the bar is the specific treatment effect (red).

### Subgroup and meta-regression analyses

Subgroup analyses were carried out in order to examine the predictors of PCE and to explore reasons for heterogeneity. Some variations were observed for pain outcome, e.g. the PCE increased with longer duration of treatment, higher proportion of women, and greater number of participants (Supplementary file 2). However, meta-regression analysis confirmed that none of these factors was a predictor for pain and only duration of treatment > 4 weeks was a predictor for FIQ total (Table 3, Supplementary file 3).

**Table 3:** Meta-regression of determinants of the proportion of contextual effect (PCE) for pain

Variable	Exp(β)	SE	95% CI	*P* value
Duration of treatment (≤ 4 weeks vs. > 4 weeks)	1.47	0.40	0.84 to 2.56	0.168
Proportion of female participants (%)	1.00	0.01	0.98 to 1.02	0.886
Number of participants (< 100 vs. ≥ 100)	1.09	0.20	0.74 to 1.59	0.665
Allocation concealment (yes vs. no)	0.94	0.20	0.62 to 1.43	0.767
Blinding (none vs. patient blinding vs. double blinding)	1.04	0.15	0.78 to 1.41	0.773

*PCE* proportion of contextual effect, *CI* confidence interval, *Q* heterogeneity statistic, *I* ^2^ the variation in ES attributable to heterogeneity, *CNS* central nervous system, *FIQ* Fibromyalgia Impact Questionnaire

Additional subgroup analysis was undertaken for FDA-approved treatments for fibromyalgia, specifically duloxetine, pregabalin, and milnacipran (www.fda.gov). For pain outcomes, the PCE was 0.66 (95% CI 0.60 and 0.73) for duloxetine, 0.58 (95% CI 0.56 to 0.60) for pregabalin, and 0.81 (95% CI 0.70 to 0.94) for milnacipran. For FIQ score, the PCE was 0.66 (95% CI 0.59 to 0.74) for duloxetine, 0.70 (95% CI 0.68 to 0.73) for pregabalin, and 0.65 (95% CI 0.58 to 0.72) for milnacipran. Forest plots are presented in Supplementary file 4.

## Discussion

This is the first study to emphasise the total treatment effect and the proportion of this effect that may be attributable to placebo/contextual effects using the RCT data in fibromyalgia. The study found that the majority of the total treatment effect was contextual (60% for pain, 57% for FIQ, and 63% for fatigue, respectively). This suggests that people with fibromyalgia benefit more from the contextual effects of a treatment than from the specific treatment effect. More interestingly, the proportion varied considerably between treatments, ranging from 44% for electromagnetic therapy and analgesics to 81% for milnacipran (Fig. 3). It is important to recognise that many factors in routine clinical practice influence the magnitude of placebo/contextual response such as patient’s expectations and knowledge of being treated (‘placebo analgesia’ [15]), patient education and patient-doctor interactions (attentive and optimistic ‘positive consultation’, holistic assessment, and desire to follow-up) [16].

This study presents a novel efficacy hierarchy for different treatments based on the total treatment effect and presents the proportion of the total treatment that is attributable to the specific and contextual effects (Fig. 3). This hierarchy has several *clinical implications*: [1] it informs practitioners about the best and the least effective treatments in fibromyalgia according to overall treatment benefits, which is more likely to accord with their experience in clinical practice than a hierarchy based on specific treatment effects [2]; it helps practitioners to appreciate how important contextual effects are in clinical care for people with fibromyalgia and suggests a way to improve current healthcare delivery through optimisation of modifiable contextual factors while waiting for new ‘stronger’ treatments with high specific effect to be developed [3]; the hierarchy highlights the areas that need further research, such as exercise and other physical treatments, where contextual effect cannot be calculated from RCTs because of the lack of a placebo group due to difficulties in blinding. In addition, this study has found that the PCE increases with the duration of treatment (for pain) and sample size (for FIQ). These data may prove useful firstly for trialists with respect to future design of trials in fibromyalgia, and secondly for clinicians when they manage the disease in clinical practice.

### Comparison with other studies

The findings are similar to a meta-analysis of RCTs for osteoarthritis where 75% of treatment effects are explained by contextual effects [6]. The current meta-analysis concurs that the majority of benefits observed in patients are attributed to contextual effects rather than the specific treatment effects. This is not unexpected as both fibromyalgia and osteoarthritis are characterised by chronic pain, restricted function and fatigue, and often show some commonalities in terms of central pain sensitisation [17]. The overall difference in PCE for pain outcomes (60 vs. 75%) between these two conditions may reflect the nature of the disease in that fibromyalgia is often more challenging and difficult to treat than osteoarthritis.

### Limitations of study

There are several caveats to this study. Firstly, like other meta-analyses, heterogeneity may affect the outcomes. This is especially true for the primary outcome of pain where the studies collected were highly heterogeneous (*I* ^2^ = 99%). Although subgroup analysis was carried out by treatment, high heterogeneity still existed in some treatments such as electromagnetic therapy (Fig. 2). This may be due to the heterogeneity of patients involved in the trials, although the majority of trials in this meta-analysis used the ACR criteria for diagnosis. We were unable to undertake a subgroup analysis based on different diagnostic criteria or subsets so whether PCE varies according to different diagnoses or subsets of fibromyalgia remains to be investigated. Secondly, the number of trials available in each treatment is different and ranges from 2 to 16; hence, the total number of patients involved in each category varied considerably (Fig. 2). Moreover, all studies for electromagnetic therapy or acupuncture were less than 100 participants in size. For small studies, it is not possible to get reasonable estimates of the treatment effect and the proportion that may be attributable to the context. Further, larger trials are needed to stabilise the estimates. Thirdly, apart from duration of treatment, we did not find other predictors of PCE. This is not surprising as we did not have individual patient data to explore the differences in outcome between individual participants, only the differences between trials. Also, we have no information on factors that influence expectancy, which has an important impact on the magnitude of placebo and contextual response. For example, we do not know the extent to which the treatment was explained to participants and whether the information was given in a positive way. Furthermore, we have no information on the illness perceptions and anxiety levels of each participant, whether they have a catastrophizing or positive attitude, or what expectations of treatment benefit they had. It can be assumed that such factors will contribute to participant expectancy and placebo/contextual response and that the overall experience and expectancy will differ between participants receiving the same treatment, even within the same trial. Fourthly, only positive placebo effects were counted in PCE. Negative placebo effects, e.g. nocebo effects, were excluded from the analysis because of inability to log-transform negative results. This may overestimate the contextual effect, but not PCE as the latter is a ratio between placebo and treatment groups where nocebo effects, if there are any, were excluded from both groups under the assumption that treatment consists of active ingredient and placebo. Furthermore, the results have not been compared to studies with a no-treatment group. Therefore, it has been assumed that all the improvements in the placebo group are contextual. Some spontaneous improvement may be due to regression to mean, natural disease fluctuation, or artefact. However, because a proportion was calculated, these spontaneous, non-treatment-related effects should be experienced equally in the active and placebo arms. Under the assumption of the multiplicative model, these spontaneous effects will be cancelled [10].

### Conclusion and future research

This study found that a large amount of the treatment effect in fibromyalgia RCTs is attributable to contextual response. Further research needs to be done into what influences the magnitude of this response. With this knowledge, physicians could optimise the context in which they deliver treatment in everyday practice and this would translate into the best possible outcomes for the patient. We suggest that a new hierarchy according to the total treatment effect and the proportion attributable to contextual effects better presents the strength of treatment and will help dispel the efficacy paradox. However, larger, higher quality trials are needed to establish this hierarchy with confidence.

## Electronic supplementary material

Supplementary File 1 (DOCX 25 kb)

Supplementary File 2 (DOCX 33 kb)

Supplementary File 3 (DOCX 26 kb)

Supplementary File 4 (DOCX 489 kb)
