# Supplementary material for: Proportion of contextual effects in the treatment of fibromyalgia—a meta-analysis of randomised controlled trials
Source: Clin Rheumatol. 2017 Dec 20;37(5):1375–82. doi: 10.1007/s10067-017-3948-3 (PMC5913391; doi:10.1007/s10067-017-3948-3)
Supplement: Supplementary file 1 — (DOCX 25 kb) [file 10067_2017_3948_MOESM1_ESM.docx]

**Supplementary File 1. List of included trials**

1. Alfano, A. P., Taylor, A. G., Foresman, P. A., Dunkl, P. R., Mcconnell, G. G., Conaway, M. R. & Gillies, G. T. 2001. Static magnetic fields for treatment of fibromyalgia: A randomized controlled trial. The Journal of Alternative & Complementary Medicine, 7, 53-64.

2. Ali, A., Njike, V. Y., Northrup, V., Sabina, A. B., Williams, A.-L., Liberti, L. S., Perlman, A. I., Adelson, H. & Katz, D. L. 2009. Intravenous micronutrient therapy (myers' cocktail) for fibromyalgia: A placebo-controlled pilot study. The Journal of Alternative and Complementary Medicine, 15, 247-257.

3. Almeida, T. F., Roizenblatt, S., Benedito-Silva, A. A. & Tufik, S. 2003. The effect of combined therapy (ultrasound and interferential current) on pain and sleep in fibromyalgia. Pain, 104, 665-672.

4. Arnold, L. M., Chatamra, K., Hirsch, I. & Stoker, M. 2010a. Safety and efficacy of esreboxetine in patients with fibromyalgia: An 8-week, multicenter, randomized, double-blind, placebo-controlled study. Clinical therapeutics, 32, 1618-1632.

5. Arnold, L. M., Clauw, D., Wang, F., Ahl, J., Gaynor, P. J. & Wohlreich, M. M. 2010b. Flexible dosed duloxetine in the treatment of fibromyalgia: A randomized, double-blind, placebo-controlled trial. The Journal of rheumatology, 37, 2578-2586.

6. Arnold, L. M., Gendreau, R. M., Palmer, R. H., Gendreau, J. F. & Wang, Y. 2010c. Efficacy and safety of milnacipran 100 mg/day in patients with fibromyalgia: Results of a randomized, double‐blind, placebo‐controlled trial. Arthritis & Rheumatism, 62, 2745-2756.

7. Arnold, L. M., Goldenberg, D. L., Stanford, S. B., Lalonde, J. K., Sandhu, H., Keck, P. E., Welge, J. A., Bishop, F., Stanford, K. E. & Hess, E. V. 2007. Gabapentin in the treatment of fibromyalgia: A randomized, double‐blind, placebo‐controlled, multicenter trial. Arthritis & Rheumatism, 56, 1336-1344.

8. Arnold, L. M., Hirsch, I., Sanders, P., Ellis, A. & Hughes, B. 2012. Safety and efficacy of esreboxetine in patients with fibromyalgia: A fourteen‐week, randomized, double‐blind, placebo‐controlled, multicenter clinical trial. Arthritis & Rheumatism, 64, 2387-2397.

9. Arnold, L. M., Lu, Y., Crofford, L. J., Wohlreich, M., Detke, M. J., Iyengar, S. & Goldstein, D. J. 2004. A double‐blind, multicenter trial comparing duloxetine with placebo in the treatment of fibromyalgia patients with or without major depressive disorder. Arthritis & Rheumatism, 50, 2974-2984.

10. Arnold, L. M., Rosen, A., Pritchett, Y. L., D'souza, D. N., Goldstein, D. J., Iyengar, S. & Wernicke, J. F. 2005. A randomized, double-blind, placebo-controlled trial of duloxetine in the treatment of women with fibromyalgia with or without major depressive disorder. Pain, 119, 5-15.

11. Arnold, L. M., Russell, I. J., Diri, E., Duan, W. R., Young, J. P., Sharma, U., Martin, S. A., Barrett, J. A. & Haig, G. 2008. A 14-week, randomized, double-blinded, placebo-controlled monotherapy trial of pregabalin in patients with fibromyalgia. The Journal of Pain, 9, 792-805.

12. Babu, A. S., Mathew, E., Danda, D. & Prakash, H. 2007. Management of patients with fibromyalgia using biofeedback: A randomized control trial. Indian journal of medical sciences, 61, 455.

13. Bach, G. L. & Clement, D. B. 2007. Efficacy of farabloc as an analgesic in primary fibromyalgia. Clinical rheumatology, 26, 405-410.

14. Bell, I. R., Lewis Ii, D. A., Lewis, S. E., Schwartz, G. E., Brooks, A. J., Scott, A. & Baldwin, C. M. 2004. Eeg alpha sensitization in individualized homeopathic treatment of fibromyalgia. International Journal of Neuroscience, 114, 1195-1220.

15. Bennett, R. 1998. Disordered growth hormone secretion in fibromyalgia: A review of recent findings and a hypothesized etiology. Zeitschrift für Rheumatologie, 57, S72-S76.

16. Bennett, R. M., Kamin, M., Karim, R. & Rosenthal, N. 2003. Tramadol and acetaminophen combination tablets in the treatment of fibromyalgia pain: A double-blind, randomized, placebo-controlled study. The American journal of medicine, 114, 537-545.

17. Branco, J. C., Zachrisson, O., Perrot, S. & Mainguy, Y. 2010. A european multicenter randomized double-blind placebo-controlled monotherapy clinical trial of milnacipran in treatment of fibromyalgia. The Journal of rheumatology, 37, 851-859.

18. Carette, S., Bell, M. J., Reynolds, W. J., Haraoui, B., Mccain, G. A., Bykerk, V. P., Edworthy, S. M., Baron, M., Koehler, B. E. & Fam, A. G. 1994. Comparison of amitriptyline, cyclobenzaprine, and placebo in the treatment of fibromyalgia. Arthritis & Rheumatism, 37, 32-40.

19. Carette, S., Mccain, G. A., Bell, D. A. & Fam, A. G. 1986. Evaluation of amitriptyline in primary fibrositis. A double‐blind, placebo‐controlled study. Arthritis & Rheumatism, 29, 655-659.

20. Chappell, A. S., Bradley, L. A., Wiltse, C., Detke, M. J., D’souza, D. N. & Spaeth, M. 2008. A six-month double-blind, placebo-controlled, randomized clinical trial of duloxetine for the treatment of fibromyalgia. Int J Gen Med, 1, 91-102.

21. Clauw, D. J., Mease, P., Palmer, R. H., Gendreau, R. M. & Wang, Y. 2008. Milnacipran for the treatment of fibromyalgia in adults: A 15-week, multicenter, randomized, double-blind, placebo-controlled, multiple-dose clinical trial. Clinical therapeutics, 30, 1988-2004.

22. Colbert, A. P., Markov, M. S., Banerji, M. & Pilla, A. A. 1999. Magnetic mattress pad use in patients with fibromyalgia: A randomized double-blind pilot study. Journal of Back and Musculoskeletal Rehabilitation, 13, 19-31.

23. Deluze, C., Bosia, L., Zirbs, A., Chantraine, A. & Vischer, T. L. 1992. Electroacupuncture in fibromyalgia: Results of a controlled trial. Bmj, 305, 1249-1252.

24. Distler, O., Eich, W., Dokoupilova, E., Dvorak, Z., Fleck, M., Gaubitz, M., Hechler, M., Jansen, J. P., Krause, A. & Bendszus, M. 2010. Evaluation of the efficacy and safety of terguride in patients with fibromyalgia syndrome: Results of a twelve‐week, multicenter, randomized, double‐blind, placebo‐controlled, parallel‐group study. Arthritis & Rheumatism, 62, 291-300.

25. Finckh, A., Berner, I. C., Aubry-Rozier, B. & So, A. K.-L. 2005. A randomized controlled trial of dehydroepiandrosterone in postmenopausal women with fibromyalgia. The Journal of rheumatology, 32, 1336-1340.

26. Ginsberg, F., Mancaux, A., Joos, E., Vanhove, P. & Famaey, J.-P. 1996. A randomized placebo-controlled trial of sustained-release amitriptyline in primary fibromyalgia. Journal of Musculoskeletal Pain, 4, 37-47.

27. Gür, A., Karakoc, M., Nas, K., Cevik, R., Sarac, A. & Ataoglu, S. 2002. Effects of low power laser and low dose amitriptyline therapy on clinical symptoms and quality of life in fibromyalgia: A single-blind, placebo-controlled trial. Rheumatology international, 22, 188-193.

28. Hannonen, P., Malminiemi, K., Yli-Kerttula, U., Isomeri, R. & Roponen, P. 1998. A randomized, double-blind, placebo-controlled study of moclobemide and amitriptyline in the treatment of fibromyalgia in females without psychiatric disorder. Rheumatology, 37, 1279-1286.

29. Harris, R. E., Tian, X., Williams, D. A., Tian, T. X., Cupps, T. R., Petzke, F., Groner, K. H., Biswas, P., Gracely, R. H. & Clauw, D. J. 2005. Treatment of fibromyalgia with formula acupuncture: Investigation of needle placement, needle stimulation, and treatment frequency. Journal of Alternative & Complementary Medicine, 11, 663-671.

30. Heymann, R., Helfenstein, M. & Feldman, D. 2001. A double-blind, randomized, controlled study of amitriptyline, nortriptyline and placebo in patients with fibromyalgia. An analysis of outcome measures. Clinical and experimental rheumatology, 19, 697-702.

31. Jones, K., Burckhardt, C., Deodhar, A. A., Perrin, N., Hanson, G. & Bennett, R. 2008. A six‐month randomized controlled trial of exercise and pyridostigmine in the treatment of fibromyalgia. Arthritis & Rheumatism, 58, 612-622.

32. Kravitz, H. M., Esty, M. L., Katz, R. S. & Fawcett, J. 2006. Treatment of fibromyalgia syndrome using low-intensity neurofeedback with the flexyx neurotherapy system: A randomized controlled clinical trial. Journal of Neurotherapy, 10, 41-58.

33. Martin, D. P., Sletten, C. D., Williams, B. A. & Berger, I. H. Improvement in fibromyalgia symptoms with acupuncture: Results of a randomized controlled trial. Mayo Clinic Proceedings, 2006. Elsevier, 749-757.

34. Mease, P. J., Clauw, D. J., Gendreau, R. M., Rao, S. G., Kranzler, J., Chen, W. & Palmer, R. H. 2009. The efficacy and safety of milnacipran for treatment of fibromyalgia. A randomized, double-blind, placebo-controlled trial. The Journal of rheumatology, 36, 398-409.

35. Murakami, M., Osada, K., Mizuno, H., Ochiai, T., Alev, L. & Nishioka, K. 2015. A randomized, double-blind, placebo-controlled phase iii trial of duloxetine in japanese fibromyalgia patients. Arthritis research & therapy, 17, 1.

36. Nelson, D. V., Bennett, R. M., Barkhuizen, A., Sexton, G. J., Jones, K. D., Esty, M. L., Ochs, L. & Donaldson, C. 2010. Neurotherapy of fibromyalgia? Pain Medicine, 11, 912-919.

37. Nørregaard, J., Volkmann, H. & Danneskiold-Samstøe, B. 1995. A randomized controlled trial of citalopram in the treatment of fibromyalgia. Pain, 61, 445-449.

38. Pauer, L., Winkelmann, A., Arsenault, P., Jespersen, A., Whelan, L., Atkinson, G., Leon, T., Zeiher, B., Littlejohn, G. & Bashford, G. 2011. An international, randomized, double-blind, placebo-controlled, phase iii trial of pregabalin monotherapy in treatment of patients with fibromyalgia. The Journal of rheumatology, 38, 2643-2652.

39. Ruaro, J., Fréz, A., Ruaro, M. & Nicolau, R. 2014. Low-level laser therapy to treat fibromyalgia. Lasers in medical science, 29, 1815-1819.

40. Russell, I. J., Holman, A. J., Swick, T. J., Alvarez-Horine, S., Wang, Y. G., Guinta, D. & Group, S. O.-F. S. 2011. Sodium oxybate reduces pain, fatigue, and sleep disturbance and improves functionality in fibromyalgia: Results from a 14-week, randomized, double-blind, placebo-controlled study. Pain, 152, 1007-1017.

41. Russell, I. J., Mease, P. J., Smith, T. R., Kajdasz, D. K., Wohlreich, M. M., Detke, M. J., Walker, D. J., Chappell, A. S. & Arnold, L. M. 2008. Efficacy and safety of duloxetine for treatment of fibromyalgia in patients with or without major depressive disorder: Results from a 6-month, randomized, double-blind, placebo-controlled, fixed-dose trial. Pain, 136, 432-444.

42. Russell, I. J., Vipraio, G. A., Michalek, J. E., Craig, F. E., Kang, Y.-K. & Richards, A. B. 1999. Lymphocyte markers and natural killer cell activity in fibromyalgia syndrome: Effects of low-dose, sublingual use of human interferon-alpha. Journal of interferon & cytokine research, 19, 969-978.

43. Short, E. B., Borckardt, J. J., Anderson, B. S., Frohman, H., Beam, W., Reeves, S. T. & George, M. S. 2011. Ten sessions of adjunctive left prefrontal rtms significantly reduces fibromyalgia pain: A randomized, controlled pilot study. Pain, 152, 2477-2484.

44. Spaeth, M., Bennett, R. M., Benson, B. A., Wang, Y. G., Lai, C. & Choy, E. H. 2012. Sodium oxybate therapy provides multidimensional improvement in fibromyalgia: Results of an international phase 3 trial. Annals of the rheumatic diseases, annrheumdis-2011-200418.

45. Spath, M., Stratz, T., Neeck, G., Kotter, I., Hammel, B., Amberger, C. C., Haus, U., Farber, L., Pongratz, D. & Muller, W. 2004. Efficacy and tolerability of intravenous tropisetron in the treatment of fibromyalgia. Scand J Rheumatol, 33, 267-270.

46. Staud, R., Lucas, Y. E., Price, D. D. & Robinson, M. E. 2015. Effects of milnacipran on clinical pain and hyperalgesia of patients with fibromyalgia: Results of a 6-week randomized controlled trial. J Pain, 16, 750-759.

47. Stival, M., Saray, R., Cavalheiro, P. R., Stasiak, C., Galdino, D. T., Hoekstra, B. E. & Schafranski, M. D. 2014. Acupuncture in fibromyalgia: A randomized, controlled study addressing the immediate pain response. Revista brasileira de reumatologia, 54, 431-436.

48. Sutbeyaz, S. T., Sezer, N., Koseoglu, F. & Kibar, S. 2009. Low-frequency pulsed electromagnetic field therapy in fibromyalgia: A randomized, double-blind, sham-controlled clinical study. The Clinical journal of pain, 25, 722-728.

49. Vaerøy, H., Abrahamsen, A., Førre, Ø. & Kåss, E. 1989. Treatment of fibromyalgia (fibrositis syndrome): A parallel double blind trial with carisoprodol, paracetamol and caffeïne (somadril comp®) versus placebo. Clinical rheumatology, 8, 245-250.

50. Vergne‐Sallel, P., Dufauret‐Lombardl, C., Bonnetl, C., Simonl, A., Trèvesl, R., Bonnabaul, H. & Bertinl, P. 2011. A randomised, double‐blind, placebo‐controlled trial of dolasetron, a 5‐hydroxytryptamine 3 receptor antagonist, in patients with fibromyalgia. European Journal of Pain, 15, 509-514.

51. Wolfe, F., Cathey, M. & Hawley, D. 1994. A double-blind placebo controlled trial of fluoxetine in fibromyalgia. Scandinavian journal of rheumatology, 23, 255-259.
