# Supplementary material for: Proportion of contextual effects in the treatment of fibromyalgia—a meta-analysis of randomised controlled trials
Source: Clin Rheumatol. 2017 Dec 20;37(5):1375–82. doi: 10.1007/s10067-017-3948-3 (PMC5913391; doi:10.1007/s10067-017-3948-3)
Supplement: Supplementary file 2 — (DOCX 33 kb) [file 10067_2017_3948_MOESM2_ESM.docx]

# Supplementary File 2.

## *Table 1. Subgroup analysis for pain*

|  | Number of studies | Number of participants | PCE | 95% CI | Q | $\mathbf{I}^{\mathbf{2}}$, % |
| --- | --- | --- | --- | --- | --- | --- |
| Duration of treatment |  |  |  |  |  |  |
| *<=4 weeks* | 10 | 433 | 0.416 | 0.314 to 0.549 | 154.30 | 94.2 |
| *> 4 weeks* | 34 | 8578 | 0.660 | 0.611 to 0.714 | 6319.28 | 99.5 |
| Proportion of female participants |  |  |  |  |  |  |
| *<85%* | 7 | 766 | 0.507 | 0.383 to 0.670 | 496.60 | 98.8 |
| *85.01-90%* | 4 | 888 | 0.546 | 0.441 to 0.676 | 211.60 | 98.6 |
| *90.01-95%* | 16 | 4264 | 0.615 | 0.560 to 0.675 | 1603.32 | 99.1 |
| *95.01-100%* | 11 | 2641 | 0.716 | 0.621 to 0.827 | 1771.57 | 99.4 |
| Number of participants |  |  |  |  |  |  |
| *<100 participants in each arm* | 30 | 1728 | 0.570 | 0.468 to 0.696 | 1192.78 | 97.7 |
| *≥100 participants in each arm* | 16 | 7369 | 0.650 | 0.592 to 0.714 | 5316.62 | 99.7 |
| Allocation concealment |  |  |  |  |  |  |
| *Yes* | 28 | 7063 | 0.607 | 0.556 to 0.662 | 5536.03 | 99.5 |
| *No* | 18 | 2034 | 0.573 | 0.491 to 0.668 | 1382.66 | 98.8 |
| Blinding |  |  |  |  |  |  |
| *Blind patient* | 8 | 2119 | 0.633 | 0.569 to 0.704 | 290.29 | 97.6 |
| *Blind patient and assessor* | 33 | 6433 | 0.594 | 0.543 to 0.649 | 6076.09 | 99.5 |
| *Open trial* | 5 | 545 | 0.508 | 0.334 to 0.772 | 168.73 | 98.2 |

**Abbreviations**: PCE – proportion of contextual effect; CI - confidence interval; Q - Heterogeneity statistic; I^2^ - the variation in ES attributable to heterogeneity.

## *Table 2. Subgroup analysis for FIQ score*

|  | Number of studies | Number of participants | PCE | 95% CI | Q | $\mathbf{I}^{\mathbf{2}}$, % |
| --- | --- | --- | --- | --- | --- | --- |
| Duration of treatment |  |  |  |  |  |  |
| *<=4 weeks* | 6 | 205 | 0.321 | 0.210 to 0.492 | 58.84 | 91.5 |
| *> 4 weeks* | 23 | 7343 | 0.602 | 0.558 to 0.650 | 3292.48 | 99.3 |
| Proportion of female participants |  |  |  |  |  |  |
| *<85%* | 4 | 535 | 0.637 | 0.491 to 0.827 | 24.68 | 87.8 |
| *85.01-90%* | 3 | 845 | 0.520 | 0.456 to 0.593 | 63.98 | 96.9 |
| *90.01-95%* | 12 | 3724 | 0.599 | 0.530 to 0.678 | 1805.23 | 99.4 |
| *95.01-100%* | 6 | 2025 | 0.651 | 0.568 to 0.746 | 341.19 | 98.5 |
| Number of participants |  |  |  |  |  |  |
| *<100 participants in each arm* | 15 | 871 | 0.506 | 0.422 to 0.607 | 298.80 | 95.3 |
| *≥100 participants in each arm* | 15 | 6709 | 0.608 | 0.557 to 0.664 | 2965.62 | 99.5 |
| Allocation concealment |  |  |  |  |  |  |
| *Yes* | 17 | 5844 | 0.551 | 0.500 to 0.607 | 3035.95 | 99.5 |
| *No* | 13 | 1736 | 0.602 | 0.537 to 0.674 | 493.77 | 97.6 |
| Blinding |  |  |  |  |  |  |
| *Blind patient* | 5 | 1384 | 0.605 | 0.564 to 0.648 | 10.82 | 63.0 |
| *Blind patient and assessor* | 22 | 5743 | 0.541 | 0.494 to 0.592 | 3389.67 | 99.4 |
| *Open trial* | 3 | 453 | 0.784 | 0.615 to 0.999 | 22.21 | 91.0 |

**Abbreviations**: PCE – proportion of contextual effect; CI - confidence interval; Q - Heterogeneity statistic; I^2^ - the variation in ES attributable to heterogeneity; CNS -Central nervous system; FIQ - Fibromyalgia Impact Questionnaire.
