# Supplementary material for: Proportion of contextual effects in the treatment of fibromyalgia—a meta-analysis of randomised controlled trials
Source: Clin Rheumatol. 2017 Dec 20;37(5):1375–82. doi: 10.1007/s10067-017-3948-3 (PMC5913391; doi:10.1007/s10067-017-3948-3)
Supplement: Supplementary file 3 — (DOCX 26 kb) [file 10067_2017_3948_MOESM3_ESM.docx]

# Supplementary File 3. Meta-regression of determinants of the proportion of contextual effect (PCE) for FIQ score

| **Variable** | **Exp(β)** | **SE** | **95% CI** | **P-value** |
| --- | --- | --- | --- | --- |
| Duration of treatment (<=4 weeks vs. > 4 weeks) | **1.71** | **0.37** | **1.09 to 2.68** | **0.022** |
| Proportion of female participants (%) | 0.99 | 0.01 | 0.98 to 1.00 | 0.158 |
| Number of participants (<100 vs. >100) | 1.13 | 0.11 | 0.91to 1.39 | 0.242 |
| Allocation concealment (yes vs. no) | 0.92 | 0.12 | 0.71 to 1.19 | 0.495 |
| Blinding (none vs. patient blinding vs. double blinding) | 0.93 | 0.07 | 0.79 to 1.09 | 0.321 |

**Abbreviations**: PCE – proportion of contextual effect; CI - confidence interval; Q - Heterogeneity statistic; I^2^ - the variation in ES attributable to heterogeneity; CNS -Central nervous system; FIQ - Fibromyalgia Impact Questionnaire.
