# Supplementary material for: Proportion of contextual effects in the treatment of fibromyalgia—a meta-analysis of randomised controlled trials
Source: Clin Rheumatol. 2017 Dec 20;37(5):1375–82. doi: 10.1007/s10067-017-3948-3 (PMC5913391; doi:10.1007/s10067-017-3948-3)
Supplement: Supplementary file 4 — (DOCX 489 kb) [file 10067_2017_3948_MOESM4_ESM.docx]

# Supplementary File 4.

***
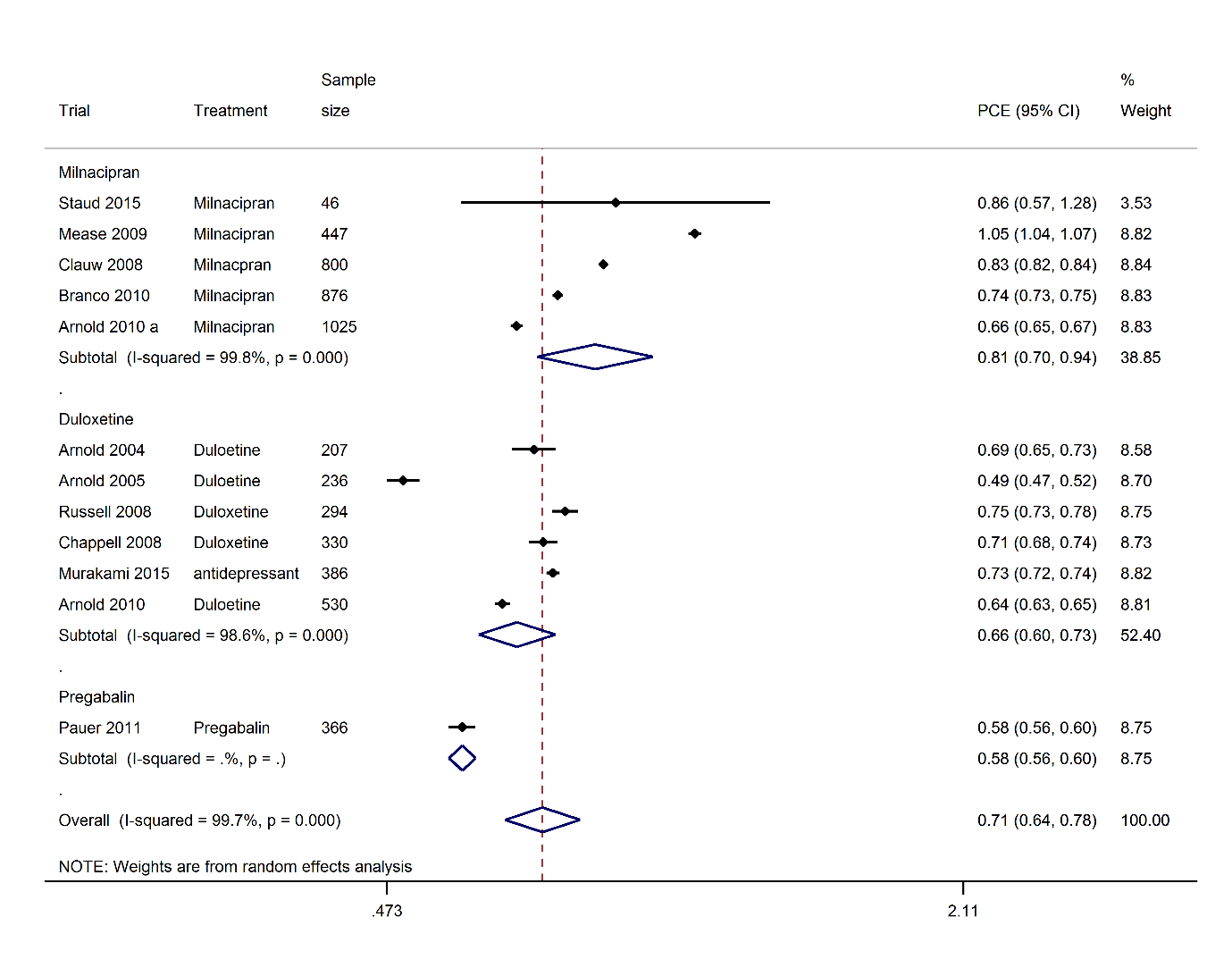
***

***Figure 1*** *Forest plot of the proportion of contextual effect (PCE) for pain in FDA-approved medications for fibromyalgia****.* Abbreviations**: PCE – proportion of contextual effect; CI - confidence interval; Q - Heterogeneity statistic; I^2^ - the variation in ES attributable to heterogeneity; CNS -Central nervous system; FIQ - Fibromyalgia Impact Questionnaire.


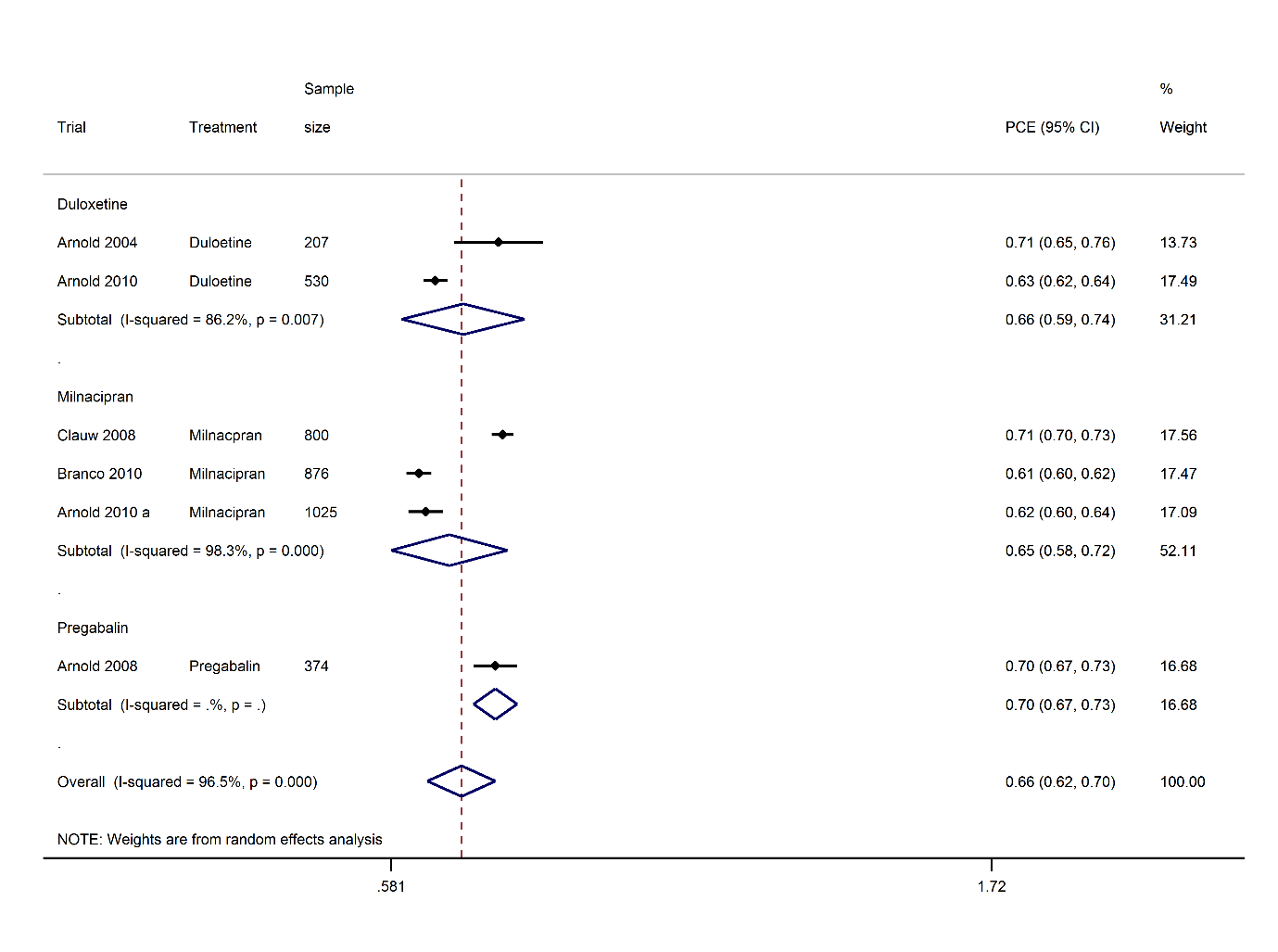


***Figure 2*** *Forest plot of the proportion of contextual effect (PCE) for FIQ-score in FDA-approved medications for fibromyalgia****.* Abbreviations**: PCE – proportion of contextual effect; CI - confidence interval; Q - Heterogeneity statistic; I^2^ - the variation in ES attributable to heterogeneity; CNS -Central nervous system; FIQ - Fibromyalgia Impact Questionnaire.
